# Stochasticity as a solution for overfitting—A new model and comparative study on non-invasive EEG prospects

**DOI:** 10.3389/fnhum.2025.1484470

**Published:** 2025-01-24

**Authors:** Yousef A. Radwan, Eslam Ahmed Mohamed, Donia Metwalli, Mariam Barakat, Anas Ahmed, Antony E. Kiroles, Sahar Selim

**Affiliations:** Center for Informatics Science (CIS), School of Information Technology and Computer Science, Nile University, Sheikh Zayed City, Giza, Egypt

**Keywords:** EEG, brain-computer interface, inner speech, machine learning, deep learning

## Abstract

The potential and utility of inner speech is pivotal for developing practical, everyday Brain-Computer Interface (BCI) applications, as it represents a type of brain signal that operates independently of external stimuli however it is largely underdeveloped due to the challenges faced in deciphering its signals. In this study, we evaluated the behaviors of various Machine Learning (ML) and Deep Learning (DL) models on a publicly available dataset, employing popular preprocessing methods as feature extractors to enhance model training. We face significant challenges like subject-dependent variability, high noise levels, and overfitting. To address overfitting in particular, we propose using “BruteExtraTree”: a new classifier which relies on moderate stochasticity inherited from its base model, the ExtraTreeClassifier. This model not only matches the best DL model, ShallowFBCSPNet, in the subject-independent scenario in our experiments scoring 32% accuracy, but also surpasses the state-of-the-art by achieving 46.6% average per-subject accuracy in the subject-dependent case. Our results on the subject-dependent case show promise on the possibility of a new paradigm for using inner speech data inspired from LLM pretraining but we also highlight the crucial need for a drastic change in data recording or noise removal methods to open the way for more practical accuracies in the subject-independent case.

## 1 Introduction

In an ever-evolving world, groundbreaking innovations like Brain-Computer Interface (BCI) technology, which enables direct communication between the brain and external devices (Wolpaw, 2007), profoundly impact individuals facing daily challenges in communication due to diverse conditions such as deafness or locked-in syndrome (Ortiz-Rosario and Adeli, 2013; Zander and Kothe, 2011; Mudgal et al., 2020). Various electroencephalogram (EEG) signals are used across different BCI applications due to their non-invasive nature, such as the P300 signal, which is utilized in the P300 speller, and motor imagery, which involves generating signals by mentally simulating the movement of different limbs (Zander and Kothe, 2011; McFarland and Wolpaw, 2017). However, the inner speech signal, which involves silently verbalizing thoughts without vocalization, known as “inner thought” or “internal voice”, remains underutilized (Nieto et al., 2022). This signal offers a substantial potential for enhancing BCI technology and usability (Nieto et al., 2022). Inner speech is not limited to limb movement, unlike motor imagery signals (Padfield et al., 2019; Park et al., 2021; Lun et al., 2022). Furthermore, it does not require visual stimuli like the P300 signal (Mudgal et al., 2020). These characteristics broaden its applications and make interactions more natural.

Despite their potential, EEG signals often have a high noise-to-signal ratio (Torquato Rollin and Buenrostro-Leiter, 2022; Lopez-Bernal et al., 2022), making it difficult to distinguish the relevant components from background brain activity caused by muscle or organ movements, eye movements, or blinks. Additionally, EEG signals are subject-dependent, meaning each individual has a unique signal pattern (Ng and Guan, 2023). This high noise and variability significantly impacts the performance of classification models. To mitigate this problem, researchers can use advanced preprocessing techniques and sophisticated classifiers. With the high noise-to-signal ratio, which is significantly higher in inner speech signals, a few more factors play a role in the challenges and difficulties of acquiring inner speech EEG signals. For instance, the absence of external stimuli. The reason this makes the desired signal acquisition complex is due to the fact that the presence of an external stimulus triggers particular neural responses, such as in the P300 and Motor Imagery signals. This phenomenon makes it easier to detect and acquire the desired signal, as observed in Medina-Juliá et al. (2020). Hence, its absence makes signal detection and acquisition difficult. Another challenge faced with this particular type of signal is its variability and difference from one individual to another (Buenrostro-Leiter and Rollin, 2022). To elaborate, in signals such as P300 and Motor Imagery, the sources of the signals are known and fixed. Accordingly, the headset's electrodes are placed in universally known places. On the other hand, the signal sources for inner speech are different from one individual to another (Montoya-Mart́ınez et al., 2021). Consequently, the electrodes placement for collecting these signals is complicated, as there is no particular pattern one can follow that will ensure this signal is accurately acquired.

To say the least, utilizing this signal will be life-changing for numerous individuals, particularly people with communication or some physical disabilities. The main advantage of inner speech is that it comes naturally to individuals, where they can simply think of the word they desire, and its respective command or use will be executed. That being said, inner speech can be incorporated in many day-to-day activities or uses for people such as navigating a wheelchair. To elaborate, when navigating a wheelchair, it would be easier to move in the desired direction by simply pronouncing it, such as saying “left” to go in that direction. That way, the process and experience will be easier and more natural. Additionally, the progression of this technology will make it possible for people with communication disabilities to talk easily and more naturally. Instead of needing to write their thoughts or have someone communicate their thoughts somehow, they will simply think of what they wish to say and it will be vocalized or represented in whatever way they wish. These examples only show a glimpse of the potential of such unique and rich technology. While its acquisition can be challenging, its advantages and possible uses make it worth the challenge and scientific effort.

To enhance the performance of inner speech EEG in BCIs, researchers have implemented various ML and DL models with different preprocessing techniques. Using the “Thinking out loud” dataset (Nieto et al., 2022), van den Berg et al. (2021) explored EEG classification with a DL approach. Firstly, they applied a band-pass filter (0.5–100 Hz) and a notch filter (50 Hz), removed artifacts using Independent Component Analysis (ICA), and downsampled the data to 254 Hz. The researchers focused on channels in the left hemisphere, and employed the EEG-Net Convolutional Neural Network (CNN) architecture. Their approach achieved an average accuracy of 29.67%, with the highest accuracy being 34.5% and the lowest 23.75% across different subjects.

Gasparini et al. (2022) explored various ML and DL models, including support vector machine (SVM), XGBoost, long short-term memory (LSTM), and bidirectional LSTM (BiLSTM). They employed preprocessing techniques such as band-pass filtering and artifact removal via Independent Component Analysis (ICA), following methods used in prior studies (van den Berg et al., 2021). Their findings highlighted the potential of SVM and XGBoost with 26.2% and 27.9% accuracy. LSTM and BiLSTM got 30.4% and 36.1%.

In another study, Merola et al. (2023) applied Random Forest, SVM, and K-Nearest Neighbor (KNN) to the same dataset (Nieto et al., 2022). The authors extracted the important features by applying several MATLAB member functions to each set of epochs. They were able to achieve a test accuracy of 27.5%, 33.9%, and 25.8% for each of the Random Forest, SVM, and KNN, respectively.

On the other hand, a similar study done by Abdulghani et al. (2024) achieved even higher results. In this study, they tested their approaches on two different datasets. The first dataset is a publicly available Spanish dataset, while the second is a 4-word English dataset developed by the authors of the study. Their approach that achieved the highest accuracy involved combining multi-wavelet analysis with SVM classification. In doing so, they were able to obtain an accuracy of 68.2% with the first dataset and 97.5% in the second one. These results are, to say the least, incredibly promising to the field, as it shows that we are slowly but surely getting closer to achieving the goal of incorporating this technology in the day-to-day life of individuals.

Across this review, some studies (van den Berg et al., 2021) reported accuracy per-subject, which we will refer to as the “subject-dependent” case. While others (Gasparini et al., 2022; Merola et al., 2023) focused on results for all subjects at the same time, which we will refer to as the “subject-independent” case.

The aim of this research is to push the boundaries of EEG-based inner speech classification through the following key activities:

- Assess and compare the performance of Machine Learning (ML) and Deep Learning (DL) models in both subject-dependent settings (where modeling is performed on individual subjects) and subject-independent settings (where modeling involves data from all subjects collectively).
- Develop and introduce a new model that surpasses existing ML models in per-subject accuracy while incorporating innovative approaches to reduce overfitting.
- Evaluate the effectiveness of six well-known preprocessing techniques for EEG data, as documented in contemporary studies.
- Thoroughly investigate the practical capabilities of non-invasive EEG for accurate and reliable classification, challenging current limitations in EEG-based BCI technologies.
  To emphasize, we aim to increase the performance and accuracy of inner speech classification to push this technology further and increase its usability. With data as sensitive as inner speech EEG and its critical uses, ensuring the performance of this technology is accurate before incorporating it in the day-to-day life of individuals who need it is essential. That being said, this study aims to push this study in this direction.

## 2 Methodology

This study assesses the performance of ML and DL models in both subject-dependent environments (where models are trained and tested on data from the same subject) and subject-independent environments (where models are trained on data from one set of subjects and tested on another). The “Thinking out Loud” dataset (Nieto et al., 2022) was utilized in this paper. A number of DL and ML models were implemented with different preprocessing techniques to optimize their performance on the dataset. The following sections outline the models and preprocessing techniques used in this analysis.

### 2.1 Dataset

The analysis focuses on the inner speech component of the “Thinking Out Loud” dataset (Nieto et al., 2022). This dataset comprises recordings from 10 subjects across four classes, Arriba (up), Abajo (down), Derecha (left), and Izquierda (right), each labeled in Spanish. The number of trials recorded varies between 45 and 60 per class for each subject, based on their availability. Overall, the dataset encompasses 559 trials per class, resulting in a total of 2236 data points. Data extraction is performed using the specified loader function from the (Nieto et al., 2022) GitHub repository to ensure accuracy and consistency in data handling.

### 2.2 Preprocessing techniques

A set of preprocessing techniques was chosen and used as a result of our research on previous work. They fall under multiple categories: independent component analysis (ICA), principal component analysis (PCA), discrete wavelet transform (DWT), and common spatial patterns (CSP). ICA and PCA were both run using all components. The CSP algorithm was used with pairwise comparison and one-versus-all comparison, each separately. One additional method that was tried was common average referencing (CAR) combined with discrete wavelet transformation. The last approach used was concatenating the outputs of all of the previous methods together: “All”. These methods were selected due to their abundant appearance in the EEG literature as effective feature extraction methods for EEG signal processing. In order to target the best performance possible, we used the best-known preprocessing techniques that were seen in the literature.

The selected preprocessing techniques aim to enhance EEG signal analysis by extracting meaningful features and reducing noise or redundancy in the data. Independent Component Analysis (ICA) is used to separate mixed signals into statistically independent components, often to isolate artifacts such as eye blinks or muscle movements from neural activity. Principal Component Analysis (PCA) reduces the dimensionality of the data by identifying orthogonal axes of maximum variance, helping to retain the most significant features while discarding less relevant information. Discrete Wavelet Transform (DWT) decomposes the EEG signal into different frequency bands, enabling the analysis of transient, non-stationary features commonly found in EEG data. Common Spatial Patterns (CSP) optimizes spatial filters to maximize the variance differences between classes, such as motor imagery tasks in brain-computer interface applications, with pairwise and one-versus-all comparisons providing flexibility in classification scenarios. Common Average Referencing (CAR) removes the common noise by re-referencing the signal to the average of all channels, improving signal-to-noise ratio, while combining it with DWT enhances the identification of localized frequency-specific features. Finally, concatenating the outputs of all these methods into a single feature set ("All") integrates the complementary strengths of each technique, capturing diverse signal characteristics and providing a comprehensive representation for further analysis. These methods are well-documented in the EEG literature as effective for feature extraction, ensuring robust preprocessing for downstream tasks.

### 2.3 Training procedure

The methodology used for this study was split into 2 categories, each split into 2 subcategories. The first split is subject-dependent experiments vs. subject-independent experiments, and then for each, we compared DL and ML models. Subject-dependent results of experiments were conducted on the data of individual subjects; this means fewer data points but more consistent data. The data was also stratified during splitting to ensure equal class distribution between train and test. Subject-independent experiments show results on the data of all 10 subjects together; the data was split so that no subject is common between train and test splits to avoid data leakage. Each of these categories is split into the ML experiments and the DL experiments. In these segments, we attempt to:

1. Find the best-performing model.
2. Find the best preprocessing or feature extraction method to improve the best-performing model.

In our ML tests, we ran data through all 20+ ML models available from the Sklearn Python library to find the best model, in addition to our new proposed model, the “BruteExtraTree” model, which involves training 1,000–2,500 ExtraTreeClassifiers with random seeds to find the best seed for test accuracy. The number of trees trained depends on the training data size, which varies with preprocessing to ensure similar training runtimes with other models. The ExtraTreeClassifier is a variant of the DecisionTreeClassifier that does not choose the best split at every splitting point and instead includes a level of stochasticity or randomness in its split selection. This model in particular is useful for combatting overfitting by forcing the model to not optimize perfectly on the data it sees and instead include a sense of variability in its split selection. Built on this, we implemented a simple algorithm to train a large number of these classifiers, each with their own random seed, and then choose the best-performing model on the development set. The models are completely independent of each other and the number of trained models, as aforementioned, was tuned according to the training set size to make the training runtime similar to its competing models. The ExtraTreeClassifier should not be mistaken for the ExtraTree**s**Classifier which is an ensemble model that provided worse performance in comparison to ours.

The ExtraTreeClassifier itself is very fast to train which leaves its 2 main limitations to be:

- Training time of multiple ExtraTreeClassifiers in relation to training set size.
- Inherent stochasticity.

The first issue has been mentioned in the previous text and can be addressed by tuning the number of training iterations. The second issue revolves around the idea that replicating the model due to its inherent stochasticity might be difficult.

The inherent stochasticity of the ExtraTreeClassifier, stemming from random feature selection and thresholding during tree construction, often results in variability across individual model runs, making it challenging to achieve reproducibility in scientific settings. However, training a large number of independent models and selecting the best-performing one on the development set leverages this randomness to effectively explore the space of possible models. Statistically, this approach increases the likelihood of identifying a model that aligns well with the data's underlying patterns, yielding consistent performance across experiments. While it does not guarantee that the selected model will generalize perfectly to unseen data, the brute-force exploration of model variability compensates for the randomness, ensuring that the approach is robust.

All ML models included normalization as a fixed preprocessing step before training in all experiments. Code for our ML pipeline and preprocessing implementations can be found in the accompanied github repository.^1^[1]

The basic procedure went as follows:

1. Split data with stratification according to given experiment scheme (subject-dependent or subject-independent).
2. Run the selected preprocessing technique (ICA, PCA, CAR+DWT, CSP, All).
3. Perform normalization of the preprocessed data.
4. Train each of the ML models separately on the data and report train and test metrics.
5. Go back to step 2 and choose the next preprocessing technique until all techniques are used.

In our DL experiments, we used Resnet18, Gated Recurrent Unit (GRU), and various EEG-specific architectures such as EEGNetv4, Deep4Net, and ShallowFBCSPNet, sourced from the Braindecode library (Schirrmeister et al., 2017), as well as deep learning-based feature extraction methods using conformers and variational autoencoders. For all experiments, we used AdamW with a learning rate of 3 × 10^−4^ and 200 epochs. This setup is the default unless stated otherwise. These parameters were chosen based on empirical evidence that they are generally effective parameters for deep learning training across a variety of fields, topics, and datasets.

The basic procedure went as follows:

1. Split data with stratification according to given experiment scheme (subject-dependent or subject-independent).
2. Put the data in the appropriate shape according to the used model: in cases of convolutional/spatial models, the data was treated as a spectrogram or image and in cases where the models were sequential, the data was put into the correct shape format that the model accepts.
3. In the case of feature extraction experiments, the data is first fed to a feature extraction model such as a conformer or convolutional variational autoencoder to train class separation and proper feature extraction.
4. The classifier model is trained on the data (coming from a feature extractor before it or as raw data) and train and test metrics are reported.
5. Go back to step 3 and run the next classifier model experiment until all configurations including feature extraction and without are complete.

All evaluations were done based on accuracy, which is detailed in Equation 1.


(1) $$\begin{array}{l} \text{Accuracy}=\frac{\text{correct predictions}}{\text{total predictions}} \end{array}$$

## 3 Results

This section presents the results of our experiments, starting with subject-dependent experiments and then subject-independent.

### 3.1 Subject-dependent experiments

#### 3.1.1 Machine learning methods

**Figure 1:**
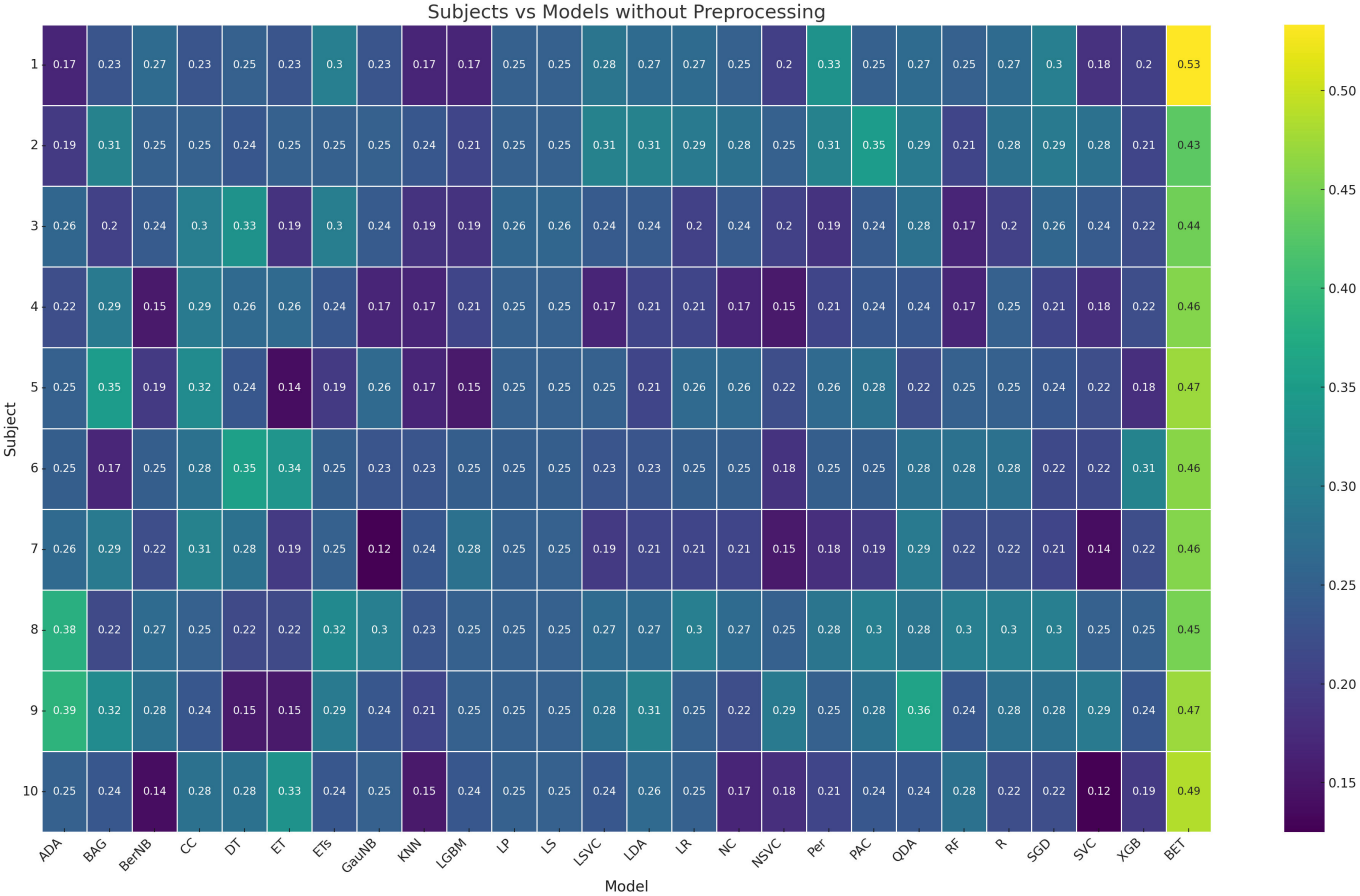
Subject vs. model test accuracy heatmap.

**Figure 2:**
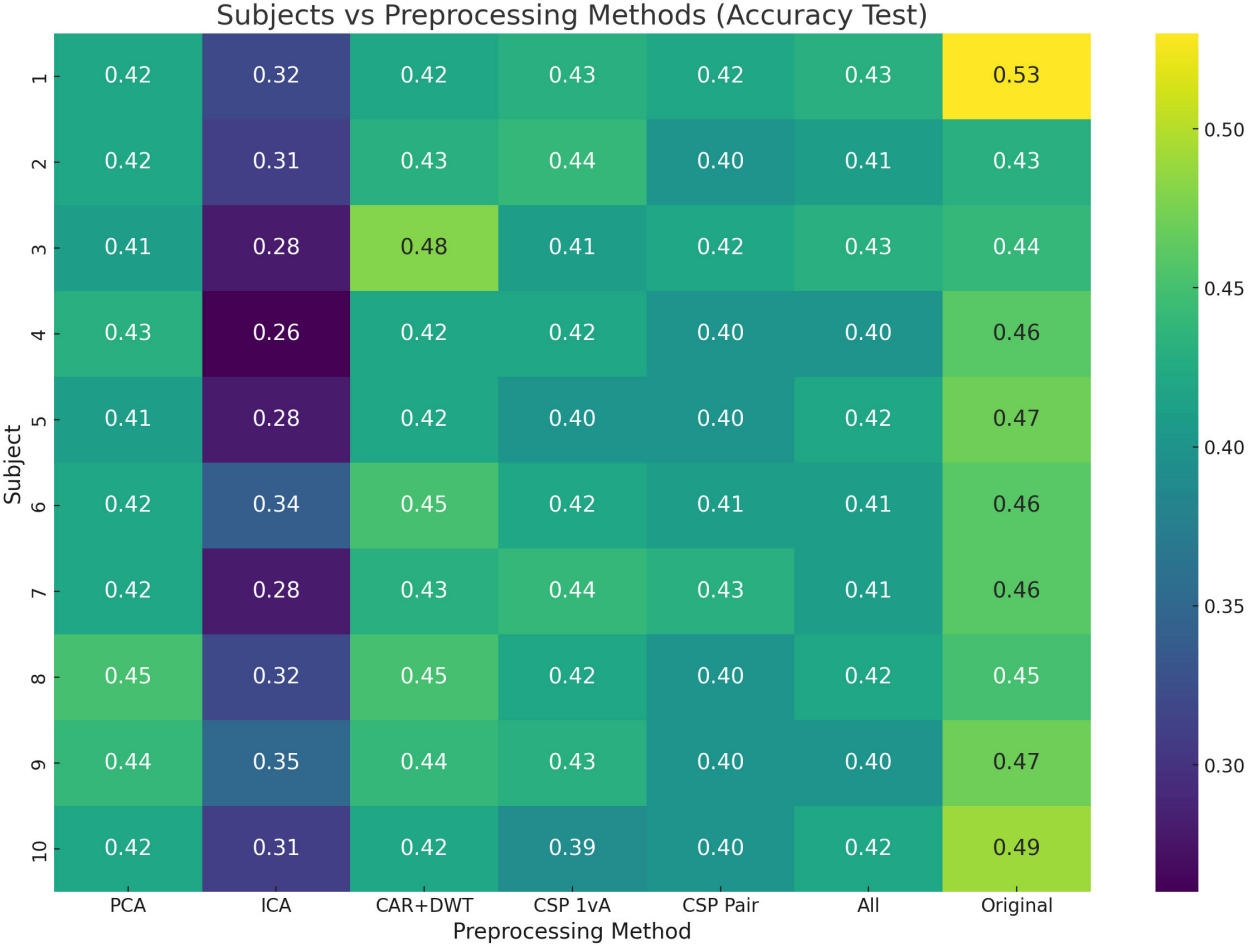
Subject vs. preprocessing test accuracy heatmap (BruteExtraTree results).

The first step is finding the best performing model on the original raw data. In Figure 1, the model accuracies of all tested models vs subjects is visualized using a heatmap. As can be seen, our proposed model BruteExtraTree significantly outperforms all other models across all subjects with an average accuracy of 46.6% in the subject dependent case. When observing the graph from the subjects axis, there seems to be no particular subject that performs notably worse than the others, neither does any one subject have notably higher scores than the rest. Across the models axis, all models always seem to drop off in performance in 1–3 subjects with moderate performance in the rest with the exception of the BruteExtraTree model. Certain models like LabelSpreading and LabelPropagation maintained consistent performance across all subjects which is significant to note. Figure 2 shows the experiments for the next step of finding the best preprocessing to improve ML model performance on the subject-dependent case. It can be seen that the preprocessing techniques do not improve the results over the raw data which is an interesting observation. ICA notably reduces accuracies across the board. These phenomena could be due to the loss of information during preprocessing which does not benefit a DecisionTree based model like the ExtraTreeClassifier. Although preprocessing methods are meant to highlight the main factors of a data sample, they do this at the cost of removing data they deem insignificant. This might be a generally useful strategy for other models but with models that make use of every feature, this can reduce performance.

#### 3.1.2 Deep learning methods

As shown in Table 1, the EEG architectures perform substantially worse than the traditional ML models. This is due to the stronger tendency of DL models to overfit, despite their specific tailoring for EEG data. The choice of learning rate also seemed very influential in these experiments. The use of a lower win rate seemed to improve performance by 3-4% in many of the cases but not all of them. The best overall performance goes to the ShallowFBCSPNet most probably due to its smaller size which lead to less overfitting overall.

**Table 1:** Performance of EEG architectures on the subjects individually.

	**EEGNetv4**	**Deep4Net**	**ShallowFBCSPNet**
Subject 1	0.25	0.27^2^	0.25^2^
Subject 2	0.27^2^	0.25	0.31^2^
Subject 3	0.25	0.30^2^	0.31^2^
Subject 4	0.25	0.25	0.33^2^
Subject 5	0.31	0.27^2^	0.31^2^
Subject 6	0.29^2^	0.25	0.29^2^
Subject 7	0.27^2^	0.31^2^	0.31^2^
Subject 8	0.32^2^	0.27	0.27
Subject 9	0.22	0.29^2^	0.39
Subject 10	0.31^2^	0.27	0.33^2^
Average accuracy	0.274	0.273	0.310

^2^3e-6 LR.

### 3.2 Subject-independent experiments

#### 3.2.1 Machine learning methods

**Figure 3:**
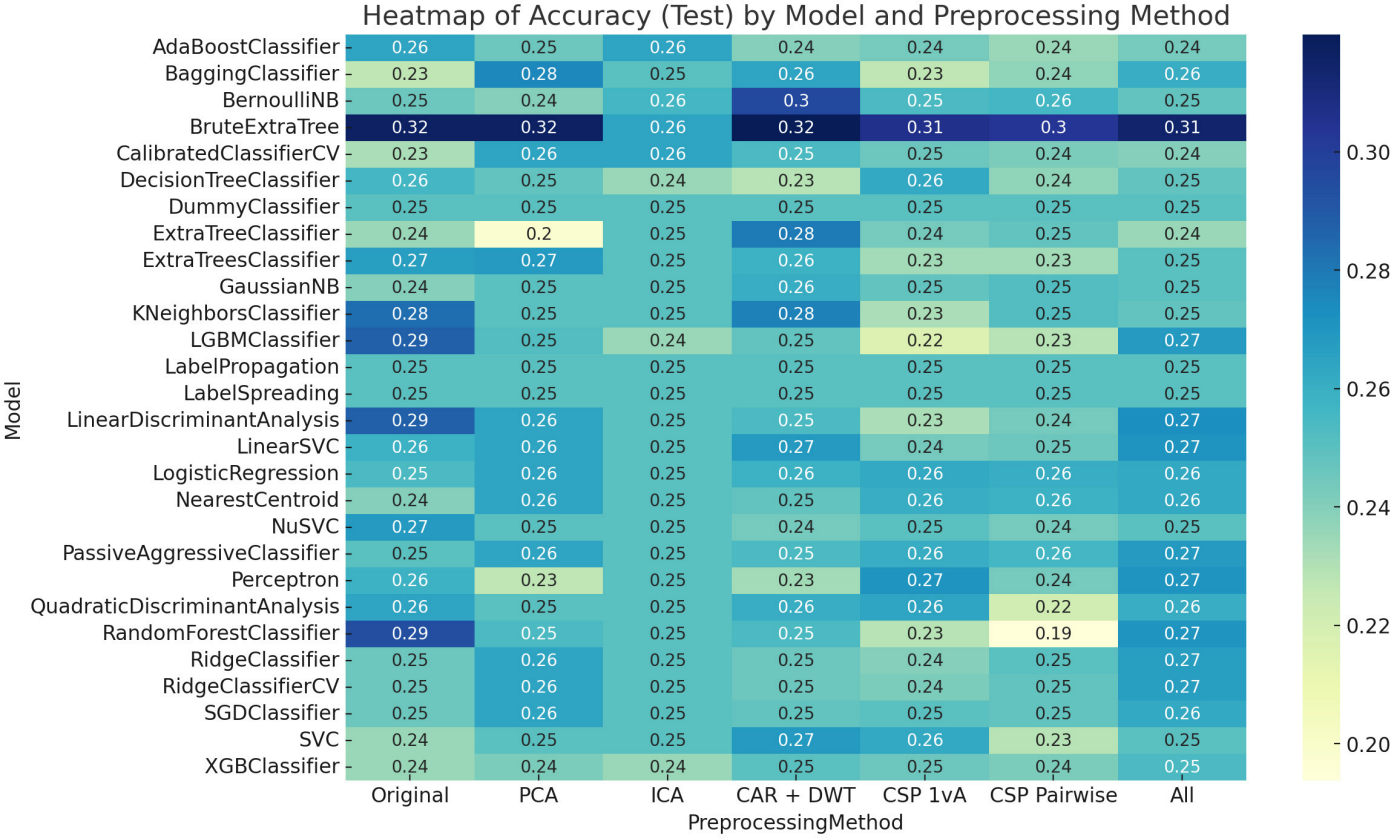
Models vs. preprocessing methods test accuracy on all subjects.

To find the best-performing model, every model was tested and implemented with every preprocessing method. Results can be seen in Figure 3. Our proposed model BruteExtraTree remains the best performing across all preprocessing methods with the highest overall accuracy and also best consistency across preprocessing techniques. To elaborate, the accuracies obtained were above 30% in 6 out of the 7 proposed preprocessing techniques, as seen in Figure 3. Next highest accuracy is BernoulliNB with CAR+DWT preprocessing with a 30% accuracy and the third highest is a three way tie between RandomForest, LinearDiscriminantAnalysis, and LGBM with a 29% accuracy, all with no preprocessing.

#### 3.2.2 Deep learning methods

The best results are summarized in Table 2. Across all experiments, Convolutional VAE with a fully connected network for classification was able to reach the highest accuracy of 32%. The second highest experiment used ML for classification instead of a FCN and showed similar results. It should be noted that the training of the feature extractors, be they conformer or variational autoencoder, showed a very smooth traditional learning curve. The focus in the training of the feature extractors was to improve class separability using losses like triplet loss and VAE loss but this did not improve results drastically compared to the literature.

**Table 2:** Deep learning methods on all subjects.

**Feature extraction**	**Training loss**	**Classifier**	**Accuracy**
None	CrossEntropy	Resnet18	0.25
None	CrossEntropy	GRU	0.24
None	CrossEntropy	EEGNetv4	0.25
None	CrossEntropy	Deep4Net	0.25
None	CrossEntropy	ShallowFBCSPNet	0.29
Conformer	TripletLoss	AdaBoost	0.27
Convolutional VAE	VAE BCE Loss	LGBM	0.28
	VAE BCE Loss + Triplet	XGB	0.31
	VAE MSE Loss + Triplet	FCN	0.32

### 3.3 Discussion and future work

The objective of this paper was to observe the behavior of different models on non-invasive inner speech EEG data. Among the experiments, accuracy was unstable and sometimes unreproducible across the experiments and models. The frequent presence of overfitting which occurred particularly with the DL models, explains why the implemented ML models generally outperformed the DL models. The highest accuracy achieved was obtained twice, the first time using a heavily complicated DL model, and the second approach with our new model, a single BruteExtraTree model, which is a much simpler ML model using a novel approach to combatting overfitting by leveraging stochasticity.

While Abdulghani et al. (2024) was able to achieve a higher accuracy than the model proposed in this paper, it is important to note that unlike the other papers mentioned, they used datasets different than the one used in this research and the other studies mentioned in the literature review. This plays an important factor since, as previously mentioned, acquiring inner speech signals is difficult since it depends on accurate electrode placement and differs from one individual to another. That being said, it is important to study the differences between how the data was acquired in the mentioned datasets and the electrode placement as they may yield more precise signals. Furthermore, it was noted that one of the factors that played a valuable role in the high accuracy they achieved was their utilization of Multi-Wavelet feature extraction techniques. While our research only utilized Discrete Wavelet Transform (DWT), we believe that combining both the approach in Abdulghani et al. (2024) and ours will have promising results, as well as investigate and find which dataset includes the most precise or cleanest inner speech signal.

Furthermore, another approach that can be implemented is using Multivariate Empirical Mode Decomposition (MEMD), which is a feature extraction technique that “decompose(s) an input EEG signal into different frequency bands called Intrinsic Mode Functions (IMFs)” (Zahra et al., 2017). The main advantage that is expected to be observed with this technique is its ability to provide high-resolution decompositions of the signals. This will enable detailed analysis of the oscillatory components without introducing artifacts or losing time-domain information. Another significant property of MEMD is that its a non-linear and data-driven approach. To elaborate, it can adaptively decompose the signals into meaningful segments or modes without making assumptions about the data structure, which makes it robust and usable with data such as EEG signals. Another approach that can be incorporated in EEG Inner Speech research is Multivariate Iterative Filtering (MIF) for feature extraction. This technique has been used with EEG signals in previous researches, such as Das and Pachori (2021). This feature extraction technique is similar to MEMD, where it is adaptive and data-driven. We believe that investigating and implemeting these approaches, as well as experimenting with different implementations is key in the advancement of this technology and will push it further, making it more usable and efficient.

Results from the literature compared to our results are seen in Table 3. It should be noted that to successfully use non-invasive inner speech EEG in practical applications, only the subject-independent environment is relevant, since we cannot possibly gather data from all possible subjects to setup subject-specific models. The general variability in data between subjects may be due to inconsistency in recording procedure (which is difficult to monitor). In order to push inner speech into application-friendly levels of accuracy, we need to either find better ways to standardize the recording procedure across subjects and also across datasets, or find new sophisticated noise removal methods that can help reduce the gap between different subjects. However, our new model does pose a new paradigm of building devices that learn from a sample of the subject data to provide tuned results for that specific subject. The idea of having a pretrained model on general inner speech EEG data and then tuning it for specific subjects can be a topic to be studied in future work.

**Table 3:** Summary of previous results and our results for subject-independent and subject-dependent cases.

**Group**	**Year**	**Model**	**Features extraction**	**Results**
Subj. indep.	van den Berg et al., 2021	EEG-Net CNN	CNN model	0.29
	Gasparini et al., 2022	SVC	XGBoost classifier	0.26
		XGBoost	XGBoost classifier	0.27
		BiLSTM	Raw	**0.36**
		LSTM	XGBoost classifier	0.30
	Merola et al., 2023	SVC	MATLAB member functions	0.33
		KNN	MATLAB member functions	0.25
		Random Forest	MATLAB member functions	0.27
	Implemented in this paper	BruteExtraTree	PCA	**0.32**
		BernoulliNB	CAR + DWT	0.30
		FCN	CVAE	**0.32**
		XGB	CVAE	0.31
Subj. dep.	van den Berg et al., 2021	EEGNet	ICA, bandpass, downsampling	**0.297**
	Implemented in this paper	BruteExtraTree	Raw	**0.466**
		ShallowFBCSPNet	Raw	0.31

Bolded results represent the highest performance achieved for each approach in the respective studies or experiments.

Our significant performance improvements in subject-dependent scenarios, compared to existing technologies, suggest that this approach may be more practical than the idealistic pursuit of subject-independent models. However, this cannot be stated with certainty. A primary challenge is acquiring a labeled sample of the subject's data for training. The required sample size remains uncertain, but with further experimentation, it may become manageable for real-life implementation.

## 4 Conclusion

This paper reviewed the performance of a wide range of models on non-invasive EEG data for inner thought classification. It utilizes the “BruteExtraTree” model, which leverages the stochastic nature of the ExtraTreeClassifier by training multiple instances with varying random seeds and selecting the optimal performer. Our findings indicate that this approach enhances classification accuracy in EEG-based inner speech recognition tasks, outperforming traditional models such as Random Forests and Support Vector Machines.

The model's inherent stochasticity poses reproducibility challenges. To address this, we recommend fine-tuning training iterations and implementing robust random seed management strategies. Future research should explore methods inspired by large language model (LLM) pretraining paradigms to reduce reliance on labeled data. Additionally, advancements in noise removal techniques and standardized EEG recording protocols are essential for improving model performance in subject-independent scenarios.

By addressing these areas, we can further the development of reliable and efficient EEG-based inner speech recognition systems, paving the way for practical applications in brain-computer interfaces and related fields.

## Data Availability

The original contributions presented in the study are included in the article/supplementary material, further inquiries can be directed to the corresponding author.

## Footnotes

^1^ https://github.com/yradwan147/InnerSpeechMLPipeline
